# Lyophilised amniotic membrane patches are a safe and effective treatment for rhegmatogenous lesions in combined tractional and rhegmatogenous retinal detachment: a prospective interventional study

**DOI:** 10.1038/s41433-024-03411-8

**Published:** 2024-10-25

**Authors:** Ángel García-Vásquez, Sergio Rojas-Juárez, Geovanni Rios-Nequis, Abel Ramirez-Estudillo

**Affiliations:** Departamento de Retina y vítreo, Fundación Hospital Nuestra Señora de la Luz, I.A.P., Ciudad de México, México

**Keywords:** Outcomes research, Retinal diseases

## Abstract

**Objectives:**

This study was performed to evaluate the safety and effectiveness of a lyophilised amniotic membrane (LAM) as a patch for the treatment of retinal breaks and to describe the structural changes at the implantation site.

**Design:**

Prospective, interventional case series study. Patients with diabetic retinopathy and combined tractional and rhegmatogenous retinal detachment.

**Methods:**

Patients were organised into 3 groups according to the number of rhegmatogenous lesions: those in group A had a single break, those in group B had two breaks, and those in group C had three or more breaks. The location of the break was also evaluated as either superior or inferior. Structural outcomes were assessed using SD-OCT during a 3-month follow-up period.

**Results:**

Of a total of 23 eyes of 23 patients, 22 (95.6%) achieved retinal repair without associated complications. Patients with 2 or fewer rhegmatogenous lesions located in the superior sector had a better anatomical result as they achieved 100% surgical success. The structural changes observed by SD-OCT over the follow-up period showed adequate adaptation of the lyophilised patch and complete closure of the rhegmatogenous lesion with no alterations in the adjacent tissue.

**Conclusions:**

The LAM patch seems to be safe and effective, as it promotes therapeutic closure of rhegmatogenous lesions without damaging the retinal architecture adjacent to the implantation site.

## Introduction

The presence of one or more rhegmatogenous lesions, also called retinal breaks, in the context of tractional retinal detachment due to advanced diabetic retinopathy could lead to a dismal prognosis and is a challenge for retinal surgeons [1]. These lesions may occur preoperatively due to the traction forces generated by the fibrous tissue on the retina or in areas of intense laser photocoagulation [2]. The formation of an incidental retinal break may occur during the removal of fibrovascular tissue during surgery [3, 4].

Laser retinopexy is sometimes ineffective for this type of lesion due to both the behaviour of chronic subretinal fluid, which is difficult to drain [5], and the rigidity of the retina associated with folds caused by proliferative components [6, 7]. Compounding the matter, the proximity of labile structures, such as the macula and optic nerve, could cause visual deterioration when a laser is applied close to them.

For these reasons, efforts to treat this type of rhegmatogenous lesion more effectively have forced the adoption of new techniques, such as the use of the internal limiting membrane (ILM) [8–10] or anterior lens capsule [11]. However, this treatment is less feasible in patients with a history of vitrectomy and peeling of the ILM, those with a history of cataract surgery or those with a clear lens. These autologous tissues could be limited for the treatment of larger or multiple rhegmatogenous lesions in complex cases.

On the other hand, human amniotic membranes (HAMs) have been used in the treatment of different vitreoretinal pathologies [12, 13]. There are 3 types of HAMs: fresh amniotic membranes, cryopreserved amniotic membranes (CAMs) and lyophilised amniotic membranes (LAMs). CAMs have been used in treating recurrent macular holes [14], paravascular retinal breaks in eyes with pathological myopia [15, 16] and maculopathy associated with optic nerve pits [17]; the results have been observed by spectral-domain optical coherence tomography (SD-OCT) and have suggested that CAMs can be used to promote anatomical closure of the break [15, 16]. Nevertheless, CAMs are not always available and affordable compared to LAMs [12]. LAMs offer the advantages of easy transportation and storage, as well as stability at room temperature [12]. This was a prospective, interventional, descriptive, case series study, in which the objective was to evaluate the safety and efficacy of a LAM (MAFIX, Top Health, Mexico) patch for the treatment of rhegmatogenous lesions in patients with combined retinal detachment and to describe the structural changes at the implantation site.

## Material and methods

### Ethical considerations

The study procedures were conducted at Fundación Hospital Nuestra Señora de la Luz I.A.P., in México City from May 2022 to March 2023 and received approval from the ethics committee in accordance with the ethical standards of the Declaration of Helsinki. Written informed consent was obtained from all patients.

### Patients

**Fig. 1:**
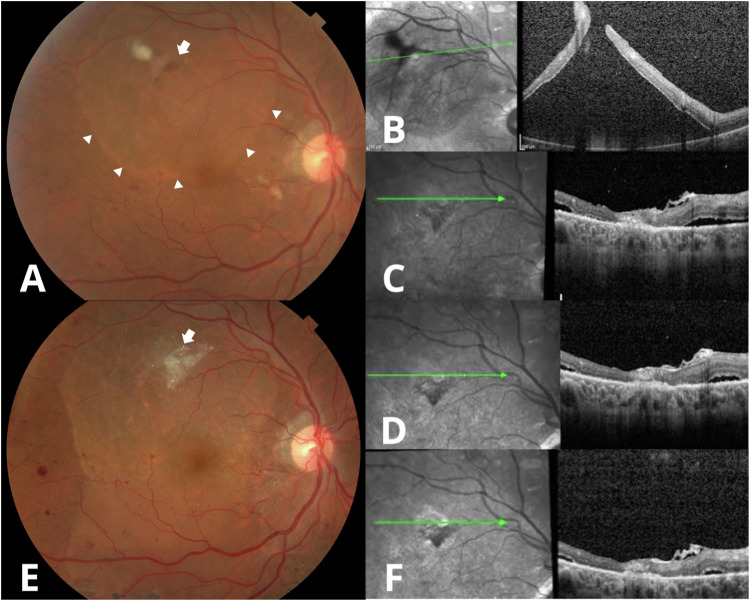
Clinical and SD-OCT evaluation of combined retinal detachment treated with LAM and SF6 tamponade. Clinical photograph of a patient with combined retinal detachment (tractional and rhegmatogenous) and a superior rhegmatogenous lesion indicated by the white arrow associated with subretinal fluid delimited by the white arrowheads prior to surgery (**A**). SD-OCT showing the rhegmatogenous lesion (**B**), structural changes 2 weeks after surgery (**C**), and changes 1 month after surgery (**D**). Clinical photograph showing the result at 3 months after phacovitrectomy and gas tamponade with sulphur hexafluoride (SF6); the LAM, indicated by the white arrow, covered the entire rhegmatogenous lesion without the presence of subretinal fluid, and there were no changes in the retinal surface close to the break (**E**). Structural changes at the 3-month follow-up (**F**).

Patients of any gender above 18 years old with diabetic retinopathy and combined retinal detachment, defined as tractional retinal detachment (TRD) associated with the presence of one or more rhegmatogenous lesions, were included. Lesions could be present prior to surgery (Fig. 1A, B) or occur incidentally during the removal of fibrovascular tissue.

All included patients were examined by refraction, biomicroscopy, tonometry, and fundus examination by indirect ophthalmoscopy before and after surgery by an SRJ retina specialist who has a high level of expertise and plenty of experience. Follow-up took place the day after surgery and at 1 week, 1 month, and 3 months post-surgery. Postsurgical outcomes were documented using fundus photographs and SD-OCT with segmentation at the site of amniotic membrane implantation. Patients with another type of retinal detachment, glaucoma, or intraocular inflammation and those included who did not comply with a minimum follow-up of 3 months were excluded.

### Surgical technique

Vitrectomy was performed through three 25-gauge ports using a standard platform (Alcon—Constellation). After central vitrectomy, the removal of fibrovascular tissue was performed by combining segmentation and delamination techniques; in complex cases, bimanual surgery was performed. Once the traction component was released, the retinal breaks were marked with diathermy, fluid-air exchange was performed, and the subretinal fluid was drained using a soft-tip cannula through either the larger rhegmatogenous lesion or the one that had a greater amount of subretinal fluid. We proceeded to dry the external orifice of the trocar cannula and the surface of the eye around the cannula with Merocel sponges. The LAM was clamped with a completely dry 25-gauge ILM clamp and then trimmed according to the break size. When introducing the membrane through the trocar cannula, folding sometimes occurred. We found that using forceps with a smaller calibre than the trocar cannula created more space and provided more freedom to mobilise the membrane towards the trocar to the vitreous cavity. If this material was not available, a second instrument of the same calibre could be used to facilitate deployment with a bimanual technique.

Once the membrane was completely deployed, it was placed over the break(s) covering the edges of the lesion in its entirety, bringing it closer to the retinal pigment epithelium (RPE). As the membrane tissue is very thin, it is difficult to distinguish the basal and stromal poles, which is why in this technique, they were considered interchangeable for placement. The surgery concluded with panretinal photocoagulation without applying a laser to the rhegmatogenous lesion. The form of endotamponade, i.e., air, gas, or silicone oil, was determined at the surgeon’s discretion. Patients were instructed to maintain a prone position for at least 3 days. All surgical procedures were performed by 2 experienced surgeons, with more than 15 years performing retinal surgery.

#### Safety evaluation

Safety was clinically evaluated and defined as a lack of adverse events or the absence of damage to the retinal tissue adjacent to the treated rhegmatogenous lesion or to the remaining intraocular structures. Efficacy was determined by evaluating SD-OCT images to assess whether complete coverage of the retinal break favouring the proximity of the edges and closure of the retinal break was achieved.

#### Surgical success

Anatomical surgical success was defined as retinal reattachment up to 3 months after surgery.

To further examine the results, patients were organised into 3 groups according to the number of rhegmatogenous lesions: those in group A had a single break, those in group B had two breaks, and those in group C had three or more breaks, as mentioned above. The greater the number of breaks, the worse the prognosis. In turn, the location of the break was determined to be superior when it was above an imaginary line drawn from the 4 to 8 o’clock meridian crossing below the inferior temporal vascular arcade, including the macular area and peripapillary region, and inferior when it was below this imaginary line, similar to the description provided by Starr in the management of inferior retinal breaks [18]. When retinal breaks coexisted in both the superior and inferior sectors, they were classified according to the sector where there was a greater number of breaks or a larger break.

#### Statistical analysis

Statistical analysis was performed using GraphPad V5.0 software. The normality of variables was tested using the Kolmogorov‒Smirnov test. The Mann‒Whitney test was used for comparisons between groups. Chi-square analysis and Fisher’s exact test were used to test differences between study group proportions using 2 × 2 and 3 × 2 contingency tables, as appropriate. *P* < 0.05 indicated statistical significance.

## Results

### Patient characteristics

A total of 23 eyes of 23 patients were included; 10 patients were men and 13 were women, and one eye was evaluated in each patient. The average patient age was 54.7 years (range: 37–66 years). All patients completed the minimum follow-up of 3 months. The average initial visual acuity was 20/250 (range: 20/50 to 20/20000), and the final average visual acuity was 20/100. Seventy-four percent of patients underwent a combined phacovitrectomy procedure, and the rest underwent only vitrectomy. Regarding endotamponade, 15 patients received 5000 cS silicone oil, 4 received room air, and 3 received gas; among the latter, 2 received SF6, and 1 received C2F6. According to the number of breaks, group A comprised 16 eyes; group B, 5 eyes; and group C, 2 eyes. Regarding the location, 19 eyes were included in the superior group, and 4 eyes were included in the inferior group.

### Surgical outcome

The aim of evaluating surgical success was to correlate the final anatomic outcomes with the impact of this novel retinopexy technique, which favours the closure of retinal breaks and therefore allows retinal reapplication.

The overall surgical success rate was 95.6% (22/23 patients). In the group analyses, both groups A and B achieved a success rate of 100%; that is, the approach was successful for those who had no more than 2 retinal breaks; however, a 50% surgical success rate was noted in group C (two eyes included), resulting in a statistically significant intergroup difference (*p* < 0.001).

Regarding location, 19 patients had superior breaks, and 4 patients had inferior breaks. The former group achieved 100% surgical success (19/19), while the latter group had a 75% success rate (3/4), resulting in a statistically significant difference between the groups (*p* < 0.045) (Table 1). In all patients, intraocular pressure remains stable within normal parameters during the follow-up.

**Table 1:** Success rates of different surgical strategies, break locations, and number of retinal breaks.

(n)	Success rate (%)	*p value*
Strategy		
Phacovitrectomy (16)	94.1	0.03*
Vitrectomy (7)	100	
Number of breaks		
Group A (16)	100	0.9**
Group B (5)	100	<0.001**
Group C (2)	50	
Location		
Superior (19)	100	0.045***
Inferior (4)	75	

*Chi square, **Fisher’s exact test, ***Mann‒Whitney.

### Structural findings

**Fig. 2:**
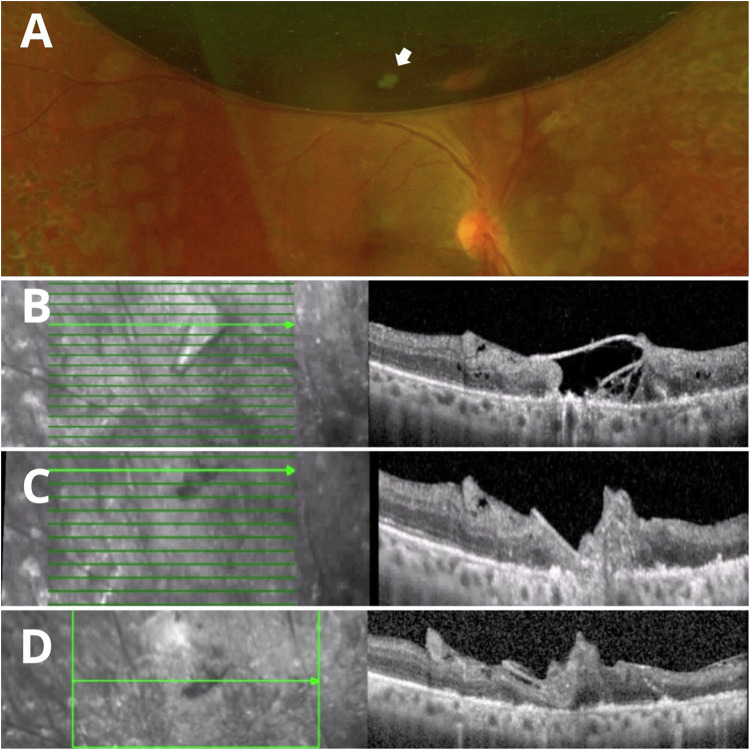
Clinical and SD-OCT evaluation of a retinal break treated with LAM and air room tamponade. Wide-field clinical photograph showing the implantation site of the LAM (white arrow) above the superior temporal vascular arcade covered by the air bubble within the first week after performing vitrectomy plus ambient air room as endotamponade (**A**). SD-OCT at the 1-week (**B**), 1-month (**C**), and 3-month follow-up (**D**) showing the structural changes the LAM undergoes over time.

**Fig. 3:**
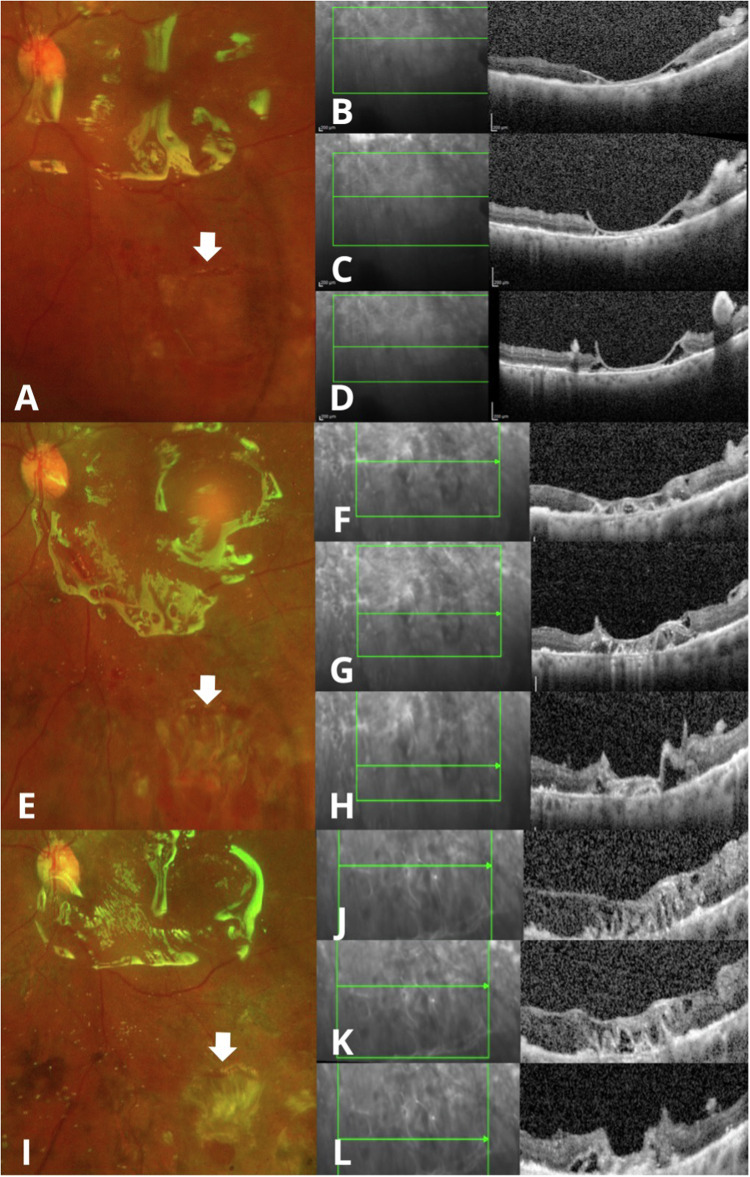
Clinical and SD-OCT evaluation of combined retinal detachment treated with LAM and silicone oil tamponade. Results in a patient with an inferior rhegmatogenous lesion (white arrow) 1 week (**A**–**D**), 1 month (**E**–**H**) and 3 months (**I**–**L**) after phacovitrectomy plus silicone oil implantation.

The findings determined by evaluating the LAM implantation site by SD-OCT were similar across patients. In the immediate postoperative period, LAM appeared well-positioned showing no evidence of misalignment fully covering the retinal break. One week postoperatively, the SD-OCT revealed the presence of a homogeneous hyperreflective band with well-defined edges over the area with the lack of continuity and in contact with the RPE, forming a bridge that connects the edges of the rhegmatogenous lesion after 1 week (Figs. 1C, 2A, B, 3A–D). At 1 month postoperatively, fundus examination revealed that the LAM remained in place, with no signs of inflammation or scarring, we noted that the proximity of the edges of the retinal break with the formation of heterogeneous hyperreflective material was associated with scattered hyperreflective foci and disorganisation of the external layers (Figs. 1D, 2C, 3E–H). By the 3-month follow-up, there was no evidence of subretinal fluid, haemorrhage, or other complications at the LAM site. SD-OCT showed dense homogeneous hyperreflective material and a decrease in hyperreflective foci associated with greater proximity to the retinal edges, disorganisation of the external layers and sub-RPE transmission defects. We found no alteration of the vitreoretinal interface or intraretinal changes aside from the LAM implantation site during follow-up. (Figs. 1E, F, 2D, 3I–L).

## Discussion

This was a prospective, interventional study to describe a unique retinopexy technique using a LAM as a patch for the treatment of rhegmatogenous lesions in patients with combined retinal detachment (tractional and rhegmatogenous). This was a novel study to describe the anatomical changes at the implantation site observed by SD-OCT during follow-up.

Our surgical technique allows effective treatment of rhegmatogenous lesions in up to 95.6% of patients, showing retinal stability during follow-up. Based on our results and experience with this new technique, we think that surgical success could be affected by the number of breaks, location, and probably the size and complexity of the retinal detachment; in the present study, we found a case of retinal redetachment in a patient in group C with inferior breaks caused by dislocation of one of the LAMs located at the inferior retina, which led to treatment failure 1 month after surgery. This patient underwent reoperation with placement of a new piece of LAM over the inferior break, plus placement of silicone oil, which, over time, allowed closure of the lesion and retinal reattachment. Despite being an isolated case, there are a few things to consider regarding this patient outcome. We know that inferior retinal breaks are often more complex to treat than superior retinal breaks; some reasons for this complexity are insufficient endotamponade and noncompliance with postoperative posturing following retinal surgery, which allow closed breaks to reopen and induce retinal redetachment. Either of these two factors in combination with gravitational forces could have caused LAM dislocation and retinopexy failure in this case. However, more studies are necessary to verify this hypothesis.

The key advantage of using LAM lies in its ability to provide anatomical closure in cases were laser retinopexy is ineffective, particularly due to the challenges posed by chronic subretinal fluid [5] and retinal rigidity caused by proliferative fold formation [6, 7] which complicate laser adhesion. Additionally, excessive laser energy during endolaser procedures can result in retinal hole formation [19]. While tamponade and positioning are still necessary, avoiding laser treatment reduces the risk of laser-induced complications, especially near sensitive structures like the macula and optic nerve, highlighting the significant benefits of LAM.

The study by Saravia [20], which reported a 100% surgical success rate in 6 patients with primary rhegmatogenous retinal detachment using LAM as a patch placed upper retinal break plus laser retinopexy, showed favourable results. This study demonstrates that using this technique for treating retinal breaks is safe and effective. Nonetheless, this study does not report the structural changes at the implantation site during the follow-up. This point could be relevant, as we experienced in our study that not every retinal break behaves the same.

To understand this behaviour, we performed SD-OCT at the implantation site of the LAM, where we could observe a well-defined homogeneous hyperreflective band attached to the surface of the retinal break in contact with the retinal edges and the RPE corresponding to the LAM. This band was present during the first 2 weeks and could be observed until the end of the monitoring period, which allowed its identification and structural analysis. Subsequently, we noted that the proximity of the retinal edges in a centripetal manner with the formation of heterogeneous hyperreflective material associated with hyperreflective foci 1 month after evolution could correspond to cell migration and the formation of glial tissue. The LAM patch constitutes a barrier that limits the exposure of the RPE to the vitreous cavity, reducing the spread of cells towards the retinal surface [21]. This could reduce the probability of developing inflammatory or fibrotic alterations that compromise retinal stability, although more studies are needed to address this hypothesis. Eventually, we found dense homogeneous hyperreflective material and a decrease in hyperreflective foci, which likely corresponds to the formation of fibrosis. However, this fibrosis was not clinically significant, as it did not affect the surrounding retinal tissue or cause any noticeable distortion. In some cases, transmission defects below the RPE, corresponding to mild atrophy, were observed, collectively indicating complete closure of the retinal break without compromising adjacent structures. These atrophic changes have also been reported in patients with a macular hole and high myopia in whom HAMs were used [22].

HAMs have unique biological characteristics due to their high concentration of proteins and growth factors that stimulate the repair and restoration of damaged tissue ^12^. It has been described that lyophilization produces lower concentrations of protein and growth factors than the other method [12, 23]. This could influence the behaviour of the membrane during follow-up by generating less inflammation and fewer fibrotic changes that alter the normal architecture of the retina adjacent to the retinal break [24].

Furthermore, this study has some limitations. Despite the small sample, compared to other reports, it constitutes the second largest series of cases in which LAMs were used for the treatment of vitreoretinal lesions. Unfortunately, analysis by group in relation to endotamponade was not possible due to the insufficient number of patients. Finally, the lack of a control group prevented assessment of whether this technique is better than standard laser retinopexy. Future investigations could compare both retinopexy techniques, including a subgroup of patients with inferior retinal breaks, which are the most difficult to treat.

## Conclusions

Overall, we concluded that the LAM patch technique appears to be safe and effective since it favours the therapeutic closure of rhegmatogenous lesions in patients with combined retinal detachment and does not damage the retinal architecture adjacent to the implantation site. The LAM patch is an additional technique that can be considered in specific cases where conventional methods may be less suitable. The changes observed by SD-OCT showed adequate adaptation of the lyophilised tissue to the implantation site.

## Summary

### What was known before

- Laser retinopexy is sometimes ineffective for this type of lesion due to both the behaviour of chronic subretinal fluid, which is difficult to drain, and the rigidity of the retina associated with folds caused by proliferative components. Compounding the matter, the proximity of labile structures, such as the macula and optic nerve, could cause visual deterioration when a laser is applied close to them.

### What this study adds

- The LAM patch seems to be safe and effective, as it promotes therapeutic closure of rhegmatogenous lesions without damaging the retinal architecture adjacent to the implantation site.

## Supplementary information

LAMPATCH Technique

eye reporting checklist

## Data Availability

The data used to support the findings of this study are available from the corresponding author upon request.
