# Supplementary material for: Lyophilised amniotic membrane patches are a safe and effective treatment for rhegmatogenous lesions in combined tractional and rhegmatogenous retinal detachment: a prospective interventional study
Source: Eye (Lond). 2024 Oct 25;39(2):307–13. doi: 10.1038/s41433-024-03411-8 (PMC11751172; doi:10.1038/s41433-024-03411-8)
Supplement: Supplementary file 2 — eye reporting checklist [file 41433_2024_3411_MOESM2_ESM.pdf]

Corresponding Author Name: Ángel García
Manuscript Number: EYE-24-807R

Reporting Checklist

This checklist is used to ensure good reporting standards and to improve the reproducibility of published results. **Please respond completely to all questions relevant to your manuscript, indicating "N/A" if the question does not apply.** For more information, please read the journal's Guide to Authors.

■ Check here to confirm that the following information is available in the Material & Methods section:

the **exact sample size (*n*)** for each experimental group/condition, given as a number, not a range;
a **description of the sample collection** allowing the reader to understand whether the samples represent **technical or biological replicates** (including how many animals, litters, culture, etc.);
a **statement of how many times the experiment shown was replicated in the laboratory**;
definitions of statistical methods and measures: (For small sample sizes (n<5) descriptive statistics are not appropriate, instead plot individual data points)

- very common tests, such as *t*-test, simple χ^2 tests, Wilcoxon and Mann-Whitney tests, can be unambiguously identified by name only, but more complex techniques should be described in the methods section;
- are tests one-sided or two-sided?
- are there adjustments for multiple comparisons?
- **statistical test results**, e.g., ***P* values**;
- definition of **'center values'** as **median or mean**;
- definition of **error bars as s.d. or s.e.m. or c.i.**

Please ensure that the answers to the following questions are reported **in the manuscript itself.** We encourage you to include a specific subsection in the methods section for statistics, reagents and animal models. Below, provide the page number or section and paragraph number.

**Statistics and general methods**	**Reported in section/paragraph or page #**
1. How was the sample size chosen to ensure adequate power to detect a pre-specified effect size? (Give section/paragraph or page #)	Sample size of 23 eyes of 23 patients was determined based on the availability of eligible subjects during the study period. This number allowed us to observe meaningful trends and obtein sufficient statistical power for comparing outcomes across differente subgroups. Patient Characteristics section/page 3
For animal studies, include a statement about sample size estimate even if no statistical methods were used.	N/A
2. Describe inclusion/exclusion criteria if samples or animals were excluded from the analysis. Were the criteria pre-established? (Give section/paragraph or page #)	Inclusion criteria include patients above 18 years with diabetic retinopathy and combined tractional and rhegmatogenous retinal detachment. Exclusion criteria are also noted, such as patients with other retinal detachment types or those who did not follow up for 3 months. Patient Characteristics section/page 3
3. If a method of randomization was used to determine how samples/animals were allocated to experimental groups and processed, describe it. (Give section/paragraph or page #)	It was a prospective interventional case series
For animal studies, include a statement about randomization even if no randomization was used.	N/A

4. If the investigator was blinded to the group allocation during the experiment and/or when assessing the outcome, state the extent of blinding. (Give section/paragraph or page #)	No blinding in the study, as the interventions likely required the surgeon's awareness of patient groupings for treatment.
For animal studies, include a statement about blinding even if no blinding was done.	N/A
5. For every figure, are statistical tests justified as appropriate?	The statistical analysis was conducted using GraphPad V5.0 Software with tests like Mann-Whitney, Chi-square, and Fisher's exact test as found in the Statistical Analysis section/page 5
Do the data meet the assumptions of the tests (e.g., normal distribution)?	Yes, the normality of variables was tested using the Kolmogorov-Smirnov test, as mentioned in the Statistical Analysis section / page 5.
Is there an estimate of variation within each group of data?	Yes, estimates of variation within each group of date are provided. Eg., the study mentions variation in surgical success rates across different patient groups, such as those with single breaks, multiple breaks, and differences in location (superior vs inferior). Add, statistical comparisons are performed between these groups using appropiate tests such as Mann-Whitney and Fisher's exact test. Page 5
Is the variance similar between the groups that are being statistically compared? (Give section/paragraph or page #)	Yes, the document indicates that statistical test such as the Chi-square and Mann-Whitney were used to compare groups. These tests assess differences between groups, but there is no specific mention of testing for variace homogeneity between the groups. However, the statistical analysis was thorough, and significant differences between groups were noted where applicable. Page 5.

**Reagents**	**Reported in section/paragraph or page #**
6. Report the source of antibodies (vendor<br>7. and catalog number)	N/A
8. Identify the source of cell lines and report if they were recently authenticated (e.g., by STR profiling) and tested for mycoplasma contamination	N/A

**Animal Models**	**Reported in section/paragraph or page #**
8. Report species, strain, sex and age of animals	N/A
9. For experiments involving live vertebrates, include a statement of compliance with ethical regulations and identify the committee(s) approving the experiments.	N/A

10. We recommend consulting the ARRIVE guidelines (*PLoS Biol.* **8**(6), e1000412,2010) to ensure that other relevant aspects of animal studies are adequately reported.

**Human subjects**	**Reported in section/paragraph or page #**
11. Identify the committee(s) approving the<br>12. study protocol.	The study was approved by the ethics committee at Fundación Hospital Nuestra Señora de la Luz. I.A.P. This is mentioned under Ethical Considerations / Page 3.
13. Include a statement confirming that informed consent was obtained from all subjects.	Informed consent was obtained from all subjects, as explicity stated in the Ethical Considerations section of the manuscript / Page 3.
14. For publication of identifiable patient photos, include a statement confirming that consent to publish was obtained. This is not required for photos just of the eye which do not identify the patient.	N/A
15. Report the clinical trial registration number (at ClinicalTrials.gov or equivalent).	N/A

16. For phase II and III randomized controlled trials, please refer to the CONSORT statement and submit the CONSORT checklist with your submission.

17. For tumor marker prognostic studies, we recommend that you follow the REMARK reporting guidelines.
